# Down-regulation of SOCS3 gene in hypothalamus attenuates diet-induced obesity in young rats

**DOI:** 10.1186/1687-9856-2013-S1-O46

**Published:** 2013-10-03

**Authors:** Jie Bian, li Zhang, xuemei Bai, Yongli Zhao, Zhengjuan Liu

**Affiliations:** 1 The Second Hospital Affiliated to Dalian Medical University, China

## Aims

Acquired childhood obesity is becoming increasingly apparent with the changes in children`s life-style and eating environment, which become a severe social and medical problem. Our previous studies have found that the leptin concentrations were high in obese children, supporting that leptin resistance is a main mechanism of childhood obesity. The suppressor of cytokine signaling 3 (SOCS3) is a negative-feedback regulator of leptin signaling involved in leptin resistance, therefore suppression of SOCS3 is a potential therapy for leptin-resistance in obesity. In the studies, we investigate whether hypothalamic silencing of SOCS3 would attenuate diet-induced obesity and leptin resistance in young rats.

## Methods

We first established hypothalamic SOCS3-deficient rats through lentiviral vector mediated RNA interference technique. The LVs expressing SOCS3-shRNA or control-shRNA were injected bilaterally into the arcuate nucleus (ARC) of five-week-old male rats, then provided a high-fat diet (HFD) to the rats. The body weight was measured weekly. After 8 weeks of the diet, the rats were killed, the serum leptin and insulin concentrations were measured by RIA, and the expressions of SOCS3 in ARC were detected by immunohistochemistry and a real time RT-PCR.

## Results

The immunostaining showed that LV-SOCS3-shRNA inhibited SOCS3 protein expression and the RNAi protocol knocked down the expression of SOCS3 mRNA by 49% compared to the controls. The rats with hypothalamic SOCS3 knockdown exhibited significant decrease in body weight gain and lower concentrations of leptin, insulin, glucose and triglyceride when exposed to the HFD.

## Conclusion

Our results provide evidence that rats with hypothalamic SOCS3 silencing are significantly protected against development of diet-induced obesity and SOCS3 is a potential target molecule for therapeutic intervention of obesity.

